# Correction: The effects of fermented vegetable consumption on the composition of the intestinal microbiota and levels of inflammatory markers in women: A pilot and feasibility study

**DOI:** 10.1371/journal.pone.0306219

**Published:** 2024-06-24

**Authors:** Amy E. Galena, Jianmin Chai, Jiangchao Zhao, Michele Bednarzyk, Doreen Perez, Judith D. Ochrietor, Alireza Jahan-Mihan, Andrea Y. Arikawa

The third author’s name is spelled incorrectly. The correct name is: Jiangchao Zhao. The correct citation is: Galena AE, Chai J, Zhao J, Bednarzyk M, Perez D, Ochrietor JD, et al. (2022) The effects of fermented vegetable consumption on the composition of the intestinal microbiota and levels of inflammatory markers in women: A pilot and feasibility study. PLoS ONE 17(10): e0275275. https://doi.org/10.1371/journal.pone.0275275.
