# Supplementary figures and images for: The effects of fermented vegetable consumption on the composition of the intestinal microbiota and levels of inflammatory markers in women: A pilot and feasibility study
Source: PLoS One. 2022 Oct 6;17(10):e0275275. doi: 10.1371/journal.pone.0275275 (PMC9536613; doi:10.1371/journal.pone.0275275)

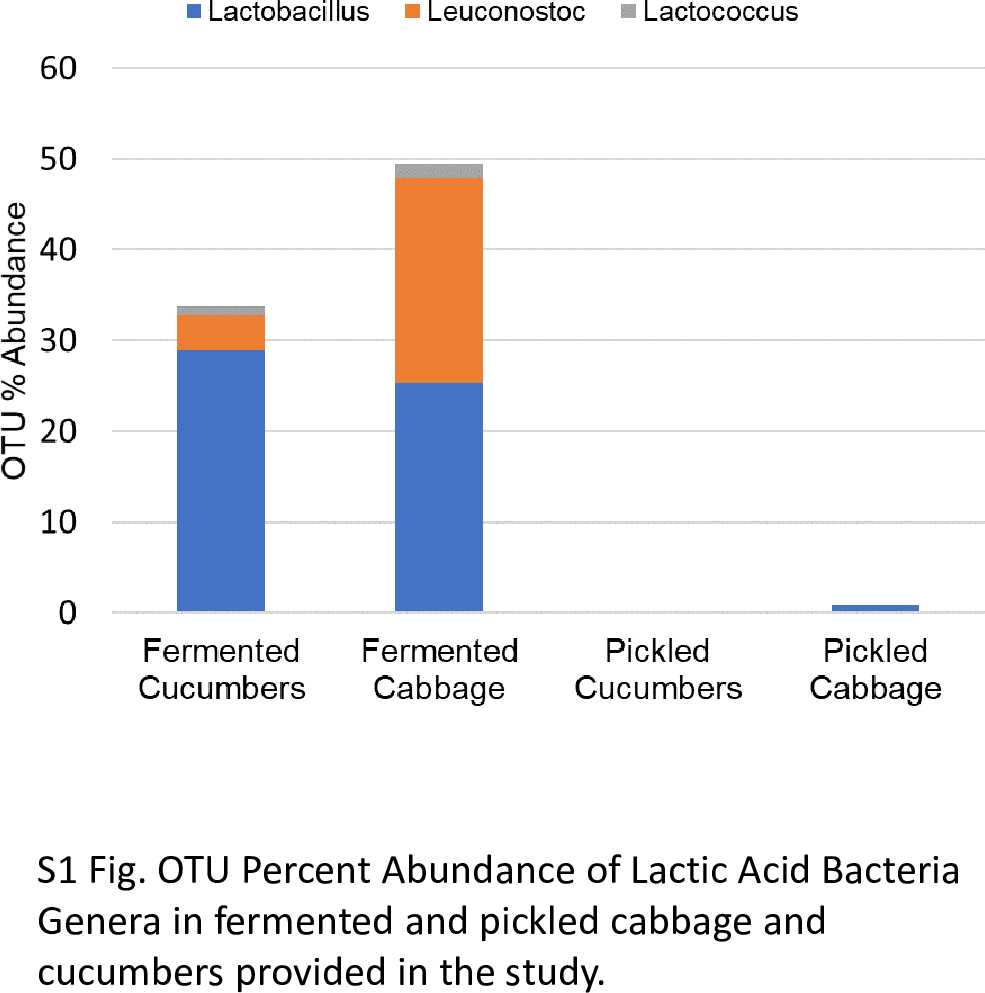

Supplement: S1 Fig — (TIF) [file pone.0275275.s001.tif]

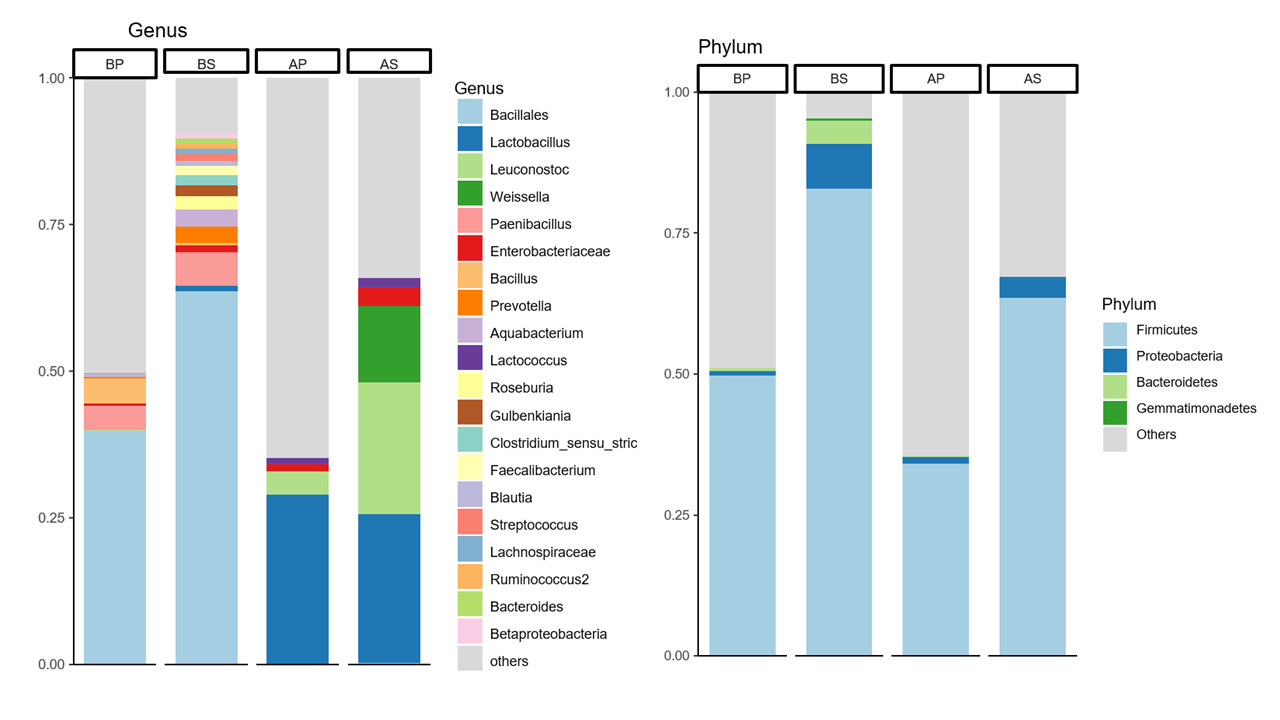

Supplement: S2 Fig — BP = pickled cucumbers, BS = pickled cabbage, AP = fermented cucumbers, AS = fermented cabbage. (TIF) [file pone.0275275.s002.tif]
