# Supplementary material for: The effects of fermented vegetable consumption on the composition of the intestinal microbiota and levels of inflammatory markers in women: A pilot and feasibility study
Source: PLoS One. 2022 Oct 6;17(10):e0275275. doi: 10.1371/journal.pone.0275275 (PMC9536613; doi:10.1371/journal.pone.0275275)
Supplement: S1 Table — (DOCX) [file pone.0275275.s003.docx]

S1 Table. Nutritional content of pickled and fermented vegetables provided in the study.

| Nutrition Facts^a^ | Pickled Vegetables  Group B | | Fermented Vegetables  Group A | |
| --- | --- | --- | --- | --- |
|  | Cucumber | Cabbage | Cucumber | Cabbage |
| Serving size, g | 28 | 30 | 36 | 36 |
| Calories, kcal | 5 | 5 | 5 | 5 |
| Total Fat, g | 0 | 0 | 0 | 0 |
| Saturated Fat, g | 0 | 0 | 0 | 0 |
| Trans Fat, g | 0 | 0 | 0 | 0 |
| Cholesterol, mg | 0 | 0 | 0 | 0 |
| Sodium, mg | 260 | 180 | 220 | 220 |
| Total Carbohydrates, g | 1 | 1 | 0 | 0 |
| Fiber, g | 0.4 | 1 | 1 | 1 |
| Total Sugars, g | 0 | 0 | 0 | 0 |
| Protein, g | 0 | 0 | 0 | 0 |

^a^Nutrition facts are based on manufacturer’s labels.
