# Supplementary material for: The effects of fermented vegetable consumption on the composition of the intestinal microbiota and levels of inflammatory markers in women: A pilot and feasibility study
Source: PLoS One. 2022 Oct 6;17(10):e0275275. doi: 10.1371/journal.pone.0275275 (PMC9536613; doi:10.1371/journal.pone.0275275)
Supplement: S2 Table — Group A: fermented vegetables, Group B: ficked vegetables, Group C: usual diet. (DOCX) [file pone.0275275.s004.docx]

S2 Table. Median values for selected dietary variables before and after the six-week intervention.

| Clinical parameter | Group A (*n*=9) | Group B (*n*=7) | Group C (*n*=5) | *P*-trend^a^ |
| --- | --- | --- | --- | --- |
| Calories (kcal/day) |  |  |  |  |
| Week 0 | 1242 (436) | 1413 (816) | 1549 (492) | 0.408 |
| Week 6 | 1284 (793) | 1185 (1069) | 1100 (449) | 0.659 |
| *P*-value^b^ | 0.859 | 0.735 | 0.043 |  |
| Carbohydrates (g/day) |  |  |  |  |
| Week 0 | 168.0 (48.2) | 190.7 (83.4) | 197.2 (117.7) | 0.717 |
| Week 6 | 181.0 (120.6) | 187.0 (95.2) | 111.3 (57.6) | 0.056 |
| *P*-value | 0.767 | 0.866 | 0.080 |  |
| Fat (g/day) |  |  |  |  |
| Week 0 | 40.0 (30.4) | 44.4 (31.9) | 53.9 (26.1) | 0.352 |
| Week 6 | 45.4 (42.7) | 36.8 (53.8) | 46.4 (35.1) | 0.997 |
| *P*-value | 0.767 | 0.866 | 0.043 |  |
| Protein (g/day) |  |  |  |  |
| Week 0 | 36.7 (24.5) | 61.7 (75.5) | 69.1 (19.8) | 0.064 |
| Week 6 | 42.4 (53.3) | 57.9 (45.8) | 56.7 (11.3) | 0.888 |
| *P*-value | 0.260 | 0.499 | 0.043 |  |
| Fiber (g/day) |  |  |  |  |
| Week 0 | 14.1 (13.2) | 19.0 (31.3) | 16.3 (11.3) | 0.965 |
| Week 6 | 17.3 (14.1) | 23.3 (16.5) | 8.1 (6.1) | 0.086 |
| *P*-value | 0.767 | 0.237 | 0.080 |  |

^a^*P*-values for the Kruskal-Wallis test for comparisons between treatment groups.

^b^*P*-values for the Wilcoxon Signed Rank test for comparisons within treatment groups.
