# Supplementary material for: The effects of fermented vegetable consumption on the composition of the intestinal microbiota and levels of inflammatory markers in women: A pilot and feasibility study
Source: PLoS One. 2022 Oct 6;17(10):e0275275. doi: 10.1371/journal.pone.0275275 (PMC9536613; doi:10.1371/journal.pone.0275275)
Supplement: S1 Protocol — (DOCX) [file pone.0275275.s006.docx]

Attachment A - Study Details

**Please note**: This attachment is required for all submission packages involving research with human participants.

# Participants

- 1. **Number of Participants**

How many participants will be enrolled? Please phrase your response as a range of participants required for your study including a minimum and maximum number required. Please differentiate between the anticipated number of participants you will contact for recruitment and the anticipated number of participants required for enrollment. For example, although you might recruit from an anticipated pool of 1000 individuals, only 150-200 participants are required for your study. If you are enrolling more than one population describe the anticipated total enrollment for each.

We will be recruiting between 30 and 42 participants. We expect to reach at least 60 potential participants through flyers posted throughout UNF and the Jacksonville area and from this pool, we expect to enroll 39 participants in order to have at least 30 participants complete the intervention. This is assuming that we will have a 20% drop out rate.

# Participant Population(s)

Describe the participant population(s) including gender, ethnicity, and age range. If any population will be specifically targeted (e.g., UNF students, minors, prisoners, UNF employees) please state as such.

Our population will be women (only) between the ages of 18 and 70 years. We are only recruiting women for this study because this was a stipulation of the funding institution. We started out recruiting women who were overweight or obese only, but due to the difficulty of recruiting this population, the inclusion criteria were expanded to include women of all weights. This project is being funded by the US Women and Girls Health Professorship, which funds research conducted to improve the health of women and girls.

# Justification of Sample Size/Data Analysis

Explain how the data will be analyzed or studied. In your response please indicate which statistical tests you anticipate use in your analyses if any. Describe how the proposed sample size is appropriate for achieving the anticipated results.

The sample size was determined based on previous studies and on budget constraints. Most of the feeding studies published to this date enrolled between 10 and 12 participants per treatment group. We are proposing 13 participants per group.

Treatment groups will be compared at both times points using analysis of variance. Changes from baseline within treatment groups in clinical and laboratory measures will be compared by paired *t* tests. Pearson's correlation coefficients will also be calculated between clinical and laboratory parameters and alterations in the intestinal microflora.

# Study Background

Outline any background information that may be relevant to your study. For example, list the research questions you are attempting to answer with this study, indicate whether there has been other research on the topic of interest, and if other research has been conducted on this topic, describe any unexpected problems involving risk or adverse events that occurred with that research. Please include any relevant background details that may be relevant to your proposed study.

Despite significant research efforts from the public and private sectors to reduce the burden of obesity, it continues to affect individuals worldwide [1]. In the United States, the prevalence of obesity among U.S. adults during 2011-2014 was 36.5% [2]. It is worthy to note, however, that during this period, the prevalence of obesity in women aged 20 to 59 years was significantly higher than in men (38.3% in women versus 34.3% in men), and if race is taken into consideration, non- Hispanic black women have the highest prevalence of obesity (56.9%) followed by Hispanic (45.7%), and non-Hispanic white women (35.5%) [2].

Over the last decade there has been an increasing interest in the relationship between the intestinal microflora and obesity as several research studies have shown an association between obesity and changes in the composition of the intestinal microflora [3-6]. For example, *Bifidobacteria*, which are regarded as probiotic bacteria that confer health benefits to the host, are found to be decreased in fecal samples of overweight and obese subjects compared to lean subjects [7]. In addition, obese subjects seem to have a lower ratio of *Firmicutes* to *Bacteroidetes*, which are some of the most abundant bacterial phyla present in the human gut [8]. Some studies suggest that the altered intestinal microflora profile of obese individuals may lead to an increase in production of lipopolysaccharide (LPS) endotoxin, which is derived from the cell wall of gram-negative bacteria in the gut (such as the *Bacteroidetes* mentioned earlier) and induces an inflammatory response by the body [9]. This may be one reason why overweight and obese individuals often present elevated levels of inflammatory markers in the blood, including LPS [10] and other more established markers of inflammation such as C- reactive protein (CRP) [11]. On the other hand, it is possible that individuals who are not obese but consume an unhealthy diet may have a different composition of the gut microflora.

The composition of the intestinal bacteria is influenced by several factors, including host genotype, age, sex, antibiotic/ medication use, disease, and diet. Changes in the intake of specific food components may lead not only to changes in the gut bacteria but may also lead to changes in gene expression of different tissues in the host, which may be directly related to more or less fat storage [12]. For example, a cross-over study looking at the effects of consumption of 4 weeks of fermented kimchi and 4 weeks of fresh kimchi in obese Korean subjects (7 male and 15 female) found significant decreases in body weight, and body fat after consumption of fermented and fresh kimchi. In addition, decreases in blood pressure, percent body fat, fasting glucose, and total cholesterol were significantly greater during the fermented kimchi phase compared with the fresh kimchi phase [13]. No side effects of kimchi consumption were reported by the authors in this study. In a parallel arm study conducted with overweight and obese Korean women, consumption of both fermented and fresh kimchi resulted in a decrease in the *Firmicutes* to *Bacteroidetes* ratio and in the relative abundance of *Bifidobacteria,* even though there were no significant differences between groups in body weight or inflammatory markers [14]. The findings from these Korean studies suggest that fresh and fermented kimchi may have different effects on intestinal bacteria and metabolism of the host. There were also no reports of side effects in this study. Unfortunately, very few studies examining the effects of fermented foods on metabolic parameters have been conducted in the U.S., likely due to the low consumption of fermented vegetables by the population. The present study was designed to investigate the effects of fermented vegetables on the composition of the gut microflora and other biological and clinical parameters associated with obesity and other chronic diseases. Our aims are:

1. To examine the effect of daily consumption of fermented vegetables compared with non-fermented vegetables for 6 weeks on markers of metabolic syndrome and inflammation in women; and
2. To examine the effect of daily consumption of fermented vegetables compared with non-fermented vegetables for 6 weeks on the profile of the gut microflora.

# Study Design

Describe your study design (e.g., longitudinal, cross-sectional, mixed-methods) and type of data to be collected (e.g., archival/secondary data, public observation, survey, interview, focus group). In your response please also describe variables of interest for your research.

This will be a parallel arm randomized controlled trial. Thirty-nine women will be randomized into one of three treatment groups: a fermented vegetable group (n=13), a non-fermented vegetable group (n=13), and a control group (n=13). The duration of the study will be 6 weeks and women in the vegetables groups will be asked to consume 1/2 cup of vegetables (140 g) per day for the entire duration of the study as well as follow their usual diet. Women randomized into the control group will be asked to follow their usual diet. Biological samples including urine, feces and blood samples will be collected at baseline and at the end of the 6-week intervention. Participants will also fill out surveys about dietary intake, physical activity, medical history, and demographics.

The variables of interest are the following: Body weight, Height, Blood pressure, Pulse, Body fat %, C-reactive protein,

Lipopolysaccharide, insulin, F2-isoprostanes, creatinine, nutrient intake, demographics, physical activity, gastrointestinal function, side effects associated with consumption of fermented vegetables, and medication use.

# Study Procedures

Describe the proposed study procedures, including the sequence and timing of all activities. In your response please also describe the data collection setting (e.g., in person, one-on-one, small groups, large groups, electric/online) and location of data collection (e.g., research lab, place of participant choosing, controlled facility). If the research involves study of existing samples/records, describe how authorization to access samples/records will be obtained.

Participants will be recruited by posting flyers throughout Jacksonville, including Women's clinics and the UNF Women’s Center and also by newspaper advertisements. We are also proposing to include an email recruitment that will be sent to a random sample of female UNF staff, faculty, and students, because recruitment has been challenging so far. We will work with UNF Institutional Research to send these emails. Interested participants will be directed to an online screening questionnaire. Those who are found eligible from the screening questionnaire will be contacted to schedule an orientation session where all study procedures will be explained in detail and the consent form will be presented.

Participants will then have the option of taking the consent form home and they will be given one to two weeks to call us back to schedule the first study visit. The orientation will take place on the 3rd floor of the Nutrition and Dietetics Department, building 39. Both baseline and follow-up visits will be in person, one-on-one and will take place in building 39A, 2nd floor, in the Nursing lab. At the first visit, participants randomized into the vegetable groups will receive one week's supply of the vegetables, and after that, vegetables will be delivered to them every other week for a total of 6 weeks. For those participants studying or working at UNF, pick up of vegetables will be available if it is convenient for them. At the end of the 6th week, participants will be asked to schedule their follow up visit at which point they will have completed the study.

The two study visits will take approximately 30 minutes and participants will be asked to schedule them in the morning because they will need to be fasting for the blood draw. Skilled phlebotomists will collect approximately 2 tablespoons of blood using sterilized needles that will be immediately disposed of following blood collection. During these visits study staff will also measure body weight, height, body fat percentage, pulse, blood pressure and collect a urine and a stool sample. The urine sample must be the first morning urine of the day of the appointment. The stool sample should be collected within 24 hours of the study visits. Participants will receive all materials necessary for the specimen collections at the orientation session.

There will be several online and paper surveys that participants will be asked to fill out following the study visits. Participants will be asked to fill those out within one week of their two study visits. The gastrointestinal function survey will be completed daily on paper and the vegetable intake side effects logs will be filled out on paper every week only by participants who are randomized into the vegetable groups. All other surveys will be completed online either via qualtrics or via a website hosted by the NCI/NIH in the case of the diet history questionnaire. For completion of this questionnaire, participants will receive a user id and password that will not contain any identifiable information.

DNA will be isolated from the stool samples using a commercial kit and placed in a polypropylene 0.5 mL screw-on tube. These samples will be shipped to the University of Arkansas for analysis of gut microflora profile. Polypropylene tubes will be wrapped in paper towels and placed inside a sealed plastic bag and these will be placed in a styrofoam box for shipment, according to FedEx packaging guidelines for clinical samples. Urine and blood samples will be aliquoted and stored in -70C freeze until analysis of the biomarkers of interest.

# Study Materials

List all study materials (e.g., survey questions, interview questions, educational materials) that will or may be used in your study. It will be necessary to submit a copy of all study materials to the UNF IRB for review and approval. Please identify material by title and submit as an independent appendix document when possible. If you plan to utilize copyrighted

information, permission from copyright owner may be necessary.

The study materials include:

- Screening questionnaire (online)
- Demographic survey
- Medical history questionnaire
- Diet history questionnaire
- Physical activity questionnaire
- Gastrointestinal function and side effects log
- Orientation slides

-Instructions on how to collect urine sample

-Instructions on how to collect stool sample

-Recruitment email script

-Acknowledgment Statement

-Response plan in case of incidental finding

# Debriefing

If any form of deception is to be used, it will be necessary to justify your reasons for including that deception. In your response please describe your proposed debriefing procedures. It will also be necessary to submit a copy of debriefing materials that will be utilized. If you will not debrief participants, please state as such in the space below.

Participants will not be debriefed.

# Benefits, Compensation, and Risk

- 1. **Participant Benefits**

Describe anticipated benefits (e.g., health screening, increased knowledge as a result of an intervention) to research participants. If participants will not benefit directly, state so here.

Benefits to participants include a final report regarding their body fat percentage, clinical measures (blood pressure, pulse), and levels of inflammatory markers. Participants in the vegetable groups will also be supplied with 1/2 cup of vegetables daily for 6 weeks.

# Societal Benefits

Describe anticipated benefits to society (e.g., added knowledge to the field of study) or a specific class of individuals (e.g., athletes or autistic children).

This proposal addresses the gap in the research with fermented foods that exists in the U.S. by examining the effects of consumption of fermented vegetables on several markers associated with obesity in women.

# Compensation

Describe compensation (e.g., extra credit toward course grade, reimbursement for travel expenses) to research participants. If participants will not be compensated, state so here. *Please note that monetary compensation may require collection of name, social security number, and address be reported to UNF controller's office.

Participants will be compensated $30 for completion of the study, $10 will be given after baseline data collection,

$10 after 4 weeks of participation and $10 after completion of follow-up data collection.

# Potential Risks to Participants

Describe the potential risks to participants and steps taken to minimize risks. Types of risks to consider include: physical, psychological, social, legal, employment, and financial.

There is a small risk of infection when blood is taken, but the risk is minimal as all needles and equipment are sterilized and the procedures are performed by skilled phlebotomists. Participants may experience some mild to moderate pain lasting a few seconds upon insertion of the needle used to draw the blood. They may also get a bruise from the blood draw. They may also experience some psychological distress when filling out some of the surveys. Intake of the fermented vegetables, particularly sauerkraut, may lead to excessive gas formation and bloating, which could cause discomfort. To minimize the effects of gas formation participants randomized to the vegetable groups will receive a combination of sauerkraut with carrots for a week, followed by pickles the following week. Both carrots and pickles are not likely to lead to gas formation. Those participants who experience more

pronounced bloating will be asked to spread the consumption of 1/2 cup of vegetables throughout the day.

# Potential Costs to Participants

Describe the potential costs to participants (e.g., invasion of privacy, time, travel) In your response please also outline how you will attempt to minimize potential costs to participants.

Participants will be asked to attend three appointments during the study. The first appointment will be an orientation session which will last around 45 minutes and the other two appointments will be the data collection visits, which should last around 30 minutes. The potential cost of this study to participants is related to time to travel to attend study visits and fill out surveys. The time commitment will be minimized by providing participants with specific instructions about parking at UNF and location of the appointment, and we also believe that the $30 incentive will help cover the costs of travel. Parking will be paid by the study for all visits.

# Risk/Benefit Analysis

Describe the ratio of risks to benefits. Risks to research participants should be justified by the anticipated benefits to the participants, the researcher's discipline, or society.

The blood draws are procedures that are widely used in the medical field and the risks are minimal, the bloating and gas formation that could result from sauerkraut consumption are normal physiological processes that can be minimized but spreading consumption throughout the day or taking over the counter medication. These risks to participants are small compared to the anticipated benefits of consumption of fermented vegetables, which may lead to decreased body inflammation and a more beneficial profile to the gut bacteria.

# Data and Safety Monitoring

- 1. **Confidentiality**

Describe procedures for protecting confidentiality of data collected and stored. Be sure to state whether any limits to confidentiality exist and identify any external agencies (e.g. study sponsor) that will have access to the data.

All study participants will be assigned an unique study ID number that will be used in all online forms and clinic forms, as well as the stored blood, urine and stool samples. There will be only one file where study IDs will be matched with identifiable information and only the principal investigator and study staff will have access to this file, which will be located in the principal investigator's computer, which is password protected.

# Data Storage, Security, and Monitoring

Describe your plan for securely storing any and all data. Be sure to identify where data will be stored, the security of this location, and how data will be monitored. ********Any breach in data safety and all unexpected problems involving risk must be reported to UNF's IRB immediately (within 3 business days or as soon as practicable).*

All data will be stored in the principal investigator's computer and backed up in an external hard drive that will be password protected and stored in a locked cabinet in the principal investigator's office. All tables containing data from surveys and analyses of biological samples will not contain any identifiable information other than the study ID for each participant. There will be only one table where study IDs will appear next to identifiable information and it will be kept in the principal investigator's computer, which is password protected. n the event that participants display higher than normal blood pressure, they will be asked to contact their primary care doctor and sign an acknowledgment statement that study staff referred them to their primary care doctor.

# Safety Monitoring:

Describe your plan for monitoring your participants and identifying any adverse effects they may experience during and (if necessary) after data collection. *Any unexpected problems involving risk must be reported to UNF's IRB immediately (within 3 business days or as soon as practicable).

Participants will be monitored every other week with respect to side effects that may result from consumption of the fermented and non-fermented vegetables. Every other week, we will deliver a two-week supply of vegetables for a total of 6 weeks. Starting on the third week, we will be collecting logs where participants will report their vegetable intake for the previous week as well as any side effects they may have experienced as a result of the intervention. These logs will be reviewed biweekly, and participants who report any type of side effect will be contacted to obtain more information. In addition, if participants display blood pressure values greater than or equal 130/80 or less than or equal to 90/60 mm Hg during the clinic visit, they will be asked to contact their primary care doctor for follow up and sign a statement acknowledging that they were informed of the altered

blood pressure readings.
