# Supplementary material for: The effects of fermented vegetable consumption on the composition of the intestinal microbiota and levels of inflammatory markers in women: A pilot and feasibility study
Source: PLoS One. 2022 Oct 6;17(10):e0275275. doi: 10.1371/journal.pone.0275275 (PMC9536613; doi:10.1371/journal.pone.0275275)
Supplement: Attachment — Submitted filename: Rebuttal Letter.docx [file pone.0275275.s007.docx]

Rebuttal Letter

PONE-D-21-29417

The effects of fermented vegetable consumption on the composition of the intestinal microbiota and levels of inflammatory markers in women: A pilot and feasibility study

Journal requirements:

When submitting your revision, we need you to address these additional requirements.

1. Please ensure that your manuscript meets PLOS ONE's style requirements, including those for file naming. The PLOS ONE style templates can be found at

<https://journals.plos.org/plosone/s/file?id=wjVg/PLOSOne_formatting_sample_main_body.pdf> and

[https://journals.plos.org/plosone/s/file?id=ba62/PLOSOne_formatting_sample_title_authors_affiliations.pdf](https://nam10.safelinks.protection.outlook.com/?url=https%3A%2F%2Fjournals.plos.org%2Fplosone%2Fs%2Ffile%3Fid%3Dba62%2FPLOSOne_formatting_sample_title_authors_affiliations.pdf&data=04%7C01%7Ca.arikawa%40unf.edu%7C6adfd102344648aa459108d9eb24ba0f%7Cdf29b2fa8929482f9dbb60ff4df224c4%7C1%7C0%7C637799366464700793%7CUnknown%7CTWFpbGZsb3d8eyJWIjoiMC4wLjAwMDAiLCJQIjoiV2luMzIiLCJBTiI6Ik1haWwiLCJXVCI6Mn0%3D%7C3000&sdata=L5T1jzx2OYMx5xF8T0xHF0Y5nfDSO09DuQHtigaegJE%3D&reserved=0)"

***I removed the spaces from the tables’ rows and used indentation formatting instead. I also added borders and shading to clearly identify the label rows.***

2. We note that the grant information you provided in the ‘Funding Information’ and ‘Financial Disclosure’ sections do not match. When you resubmit, please ensure that you provide the correct grant numbers for the awards you received for your study in the ‘Funding Information’ section.

***The ‘Funding Information’ section has been revised to match the ‘Financial Disclosure’ section.***

3. We note that you have stated that you will provide repository information for your data at acceptance. Should your manuscript be accepted for publication, we will hold it until you provide the relevant accession numbers or DOIs necessary to access your data. If you wish to make changes to your Data Availability statement, please describe these changes in your cover letter and we will update your Data Availability statement to reflect the information you provide.

***We have included in the cover letter that we made changes to the Data Availability statement and that our data will not be available at this time. We plan to make the data available once all publications related to these data are prepared.***

4. We note that you have included the phrase “data not shown” in your manuscript. Unfortunately, this does not meet our data sharing requirements. PLOS does not permit references to inaccessible data. We require that authors provide all relevant data within the paper, Supporting Information files, or in an acceptable, public repository. Please add a citation to support this phrase or upload the data that corresponds with these findings to a stable repository (such as Figshare or Dryad) and provide and URLs, DOIs, or accession numbers that may be used to access these data. Or, if the data are not a core part of the research being presented in your study, we ask that you remove the phrase that refers to these data.

***We have included a supplemental table (S2 Table) reference in the manuscript to show the data mentioned in line 276 of page 14. The supplemental table file has also been uploaded.***

5. Please include your full ethics statement in the ‘Methods’ section of your manuscript file. In your statement, please include the full name of the IRB or ethics committee who approved or waived your study, as well as whether or not you obtained informed written or verbal consent. If consent was waived for your study, please include this information in your statement as well.

***I added to page 5, line 110-111 that the University of North Florida IRB approved the study and that all study participants provided informed written consent prior to starting the study.***

[Note: HTML markup is below. Please do not edit.]

Reviewers' comments:

5. Review Comments to the Author

Please use the space provided to explain your answers to the questions above. You may also include additional comments for the author, including concerns about dual publication, research ethics, or publication ethics. (Please upload your review as an attachment if it exceeds 20,000 characters)

Reviewer #1: The authors need to clarify and address these comments.
[Title] In this study, only cabbage or cucumber

***We have revised the title to include cabbage and cucumber in place of ‘vegetable’.***

[Abstract] Not clear “feasibility”? CRP, TNF-z, LBP in blood. What about analyzing the composition of gut microflora?

***Given that regular consumption of fermented vegetables or foods, in general, is not a common practice in Western countries, an important component of this trial was to assess how feasible it would be for participants to consume fermented vegetables every day. We were interested in collecting data on how difficult or how easy it was for participants to consume the fermented vegetables regularly.***

***We have also clarified in the abstract that blood samples were collected for analysis of inflammatory markers and stool samples were collected for analysis of the gut microflora.***

[Introduction]
Line 69-70. Need more details

***At the request of the reviewer, we added the section below to this paragraph:* “For example, a high-fat diet has been linked to high abundance of the phyla *Firmicutes* and *Proteobacteria,* and low abundance of *Bacteroides* [22]*,* and a vegan diet was significantly associated with lower *Bacteroides spp., Bifidobacterium spp., Escherichia coli,* and *Enterobacteriaceae spp.* [23]. Furthermore, plant proteins such as pea protein have been shown to increase beneficial gut bacteria such as *Bifidobacteria* and *Lactobacilli*, while intake of animal protein has been found to increase the abundance of bile-tolerant bacteria, such as *Bacteroides, Alistipes,* and *Bilophila* [24]*” (lines 71-77 in the manuscript version with track changes – all references to line numbers from here on correspond to the version with track changes)***

Line 71-72. What are those high-fermented food diet?

***We added additional information about the types of fermented foods that were consumed as part of the high-fermented food diet, the sentence now reads: “A recent clinical trial found that individuals randomized to a high-fermented foods diet of six or more servings per day of fermented dairy products, fermented vegetables, and/or fermented non-alcoholic drinks showed significant increases in alpha diversity and improvements in inflammatory markers over a 10-week period [26], but more studies are needed to identify specific health benefits of various fermented foods.” (line 81)***

* [Materials & Chemicals]
Line 127. How to control them not to have fermented vegetables during the study period?

***One of the criteria for inclusion of participants into the study was that they did not regularly consume fermented vegetables. Those randomized into the control group were asked to maintain their usual dietary intake. The diet questionnaire filled out by all participants before and after the intervention provided an objective way for us to check whether those randomized into the control group consumed any fermented vegetables during the study. We did not find any red flags on the diet data. We did not feel that this was an important point to include in the paper, because our inclusion criteria stipulated that participants should not be regular consumers of fermented vegetables.***

Line 131-123. Need justification/rationale why cabbage or cucumber. Why are there two choices instead of one sample? What is the Nutrition Value of each vegetable? Different fiber (or other nutrients) content could affect the gut microbial composition.

***We really appreciate the reviewer’s thoughtful comment about the vegetables offered in the study. We added a supplemental table (S1 Table) that contains the nutrition facts of the vegetables used in the study and we also included a sentence to explain the rationale for choosing both cabbage and cucumber. The sentence reads: “It should also be noted that we chose to provide two types of vegetables for two reasons: to better represent a normal dietary pattern which includes a variety of vegetables, and to reduce the likelihood that participants would become tired of consuming one single type of vegetable during the 6-week period of the study.”(lines 144-148)***

Line 134-136. [1] Need more details of fermented cabbage/cucumbers and pickled ones- what ingredients are in it. Etc. [2] No data about taste profiles and bacterial composition.

***In addition to including a nutrition facts table in the supplemental materials mentioned earlier, we are including a supplemental figure (S1_Fig) to illustrate the abundance of lactic acid bacteria in the fermented and pickled vegetables. We also included a sentence in the methods that reads: “Microbial analysis of both fermented and pickled vegetables confirmed the presence of lactic acid bacteria in the fermented vegetables but not in the pickled vegetables (S1 Fig).” (lines 151-153)***

Line 205 & 207. Why 3 days? Why 300g?

***The duration of the study was 6 weeks, but as it often happens in clinical studies, we had to accommodate scheduling of clinic visits to participants’ availability. Sometimes, participants were not available at the exact 6-wk follow-up date and they were scheduled either a few days earlier or a few days later than the actual 6-week or 42-day mark. We chose +/- 3 days (which is equivalent to +/- 300 g of vegetables), because there were a few participants who were in the study for 39 days and who consumed the required vegetables every single day. Since they consumed all the vegetables during the entire period they were in the study, we didn’t think it was appropriate to calculate their compliance based on 42 days. Another reason for choosing 3 days is related to rounding. We felt that 5 weeks + 4 days was closer to 6 weeks.***

***We added the following sentence to this section: “The rationale for choosing ±3 days in the calculation of compliance was related to accommodating participants’ schedules for the follow-up appointments. We also felt that individuals who were able to consume 100 g of vegetables for at least 39 out of the 42 days, should be considered 100% compliant.”(lines 224-228)***

[Results]
Line 247. Fig 1. Need to mention about aim 1, 2, and 3

***Thank you for this great suggestion. We added the aims to the results as follows:***

- ***Aim 1 was added to line 279 and reads: “To assess aim 1, compliance and tolerance to the interventions were measured in groups A and B and are shown on Table 2.”***
- ***Aim 2 appears in line 295 and reads: “Aim 2 was to determine the effects of fermented vegetable consumption on markers of inflammation.”***
- ***Aim 3 appears in line 311 and reads: “Aim 3 was to determine the effects of consumption of fermented vegetables on the composition of the gut microflora, assessed via diversity and OUT abundance measures.”***

Table 1. Black-African or African America? White-Caucasian?

***We decided to go with the terms Black and White, because these are more general terms. An editorial published by JAMA in August 17, 2021 (JAMA. 2021;326(7):621-627. doi:10.1001/jama.2021.13) suggested that the term ‘Black’ be used in reporting since some individuals may identify as African while others may not. Same goes for White vs Caucasian, this is based on personal preference, but our questionnaire included more general terms such as Black or White and that is why we are reporting these races as such.***

Need statistical analysis/differences for all tables

***Table 1 is a baseline characteristics table, and according to CONSORT guidelines, reporting descriptive statistics should be the focus for pilot studies, given that the sample sizes for pilot studies are small.***

***We did add information about statistical analysis of compliance to the text. A sentence was added to the Statistical Analysis, line 233: “Compliance data were compared between groups A and B using the Mann-Whitney test.” And we also added to the results that there were no differences in compliance measures between the two groups. This was added to line 284-285. Finally, we modified Table 2 to include the p-values for the comparisons as well as the median values, which are more appropriate to report when using non-parametric tests for statistical analysis.***

Table 2. “Overall, mean (SD)” – is it correct way to see compliance? Averaging those two?

***We felt that it was appropriate to report an overall measure of compliance, given that compliance was measured in two different ways, accounting for days of participation in the study and amount of vegetables consumed.***

Line 269-270. But can we know if they were experiencing it at first but got better? 3 days out of 42 days can be shot and also how do you make sure the side effect is from fermented/pickled samples, not from other food in their diet?

***The reviewer has a great point in that it is possible that some individuals may have experienced less symptoms after the intervention compared with before the intervention. We are not able to know for sure that the side effects reported were related to the interventions, and that is why we made sure to collect side effects data from the control group. It would be interesting to look at links between prevalence of side effects and abundance of specific microbial communities in research participants, and we are beginning to explore these data further to look into a possible connection.***

Line 273. Among the three groups ◊ both before and after?

***Among the three groups relates to between group comparisons. Before and after comparisons were designated as “pairwise comparisons within each group” in line 298-299.***

Line 322-324. Decreased in most individuals in group A at week 6 compared to week 0 except on individual with high increase

***We added the words “in most individuals in group A” to be more precise. (line 352)***

Line 326. 5C ◊ any significant difference?

***Yes, the LEfSe provides data for the specific taxa that were significantly changed between the two time points. Line 354 indicates that those OTUs were ‘significantly’ more enriched at week 6 in the two groups.***

[Discussion]
Line 363. Better to show the abundance before and after to clarify the results

***Abundance of phyla before and after are shown in Figure 1A.***

Line 365-366. Need a reference. With relative abundance of 60-70%--> was this data before or after the study?

***We have added the reference and clarified the relative abundance for Firmicutes at the beginning of the study (line 394-385).***

Line 378 & 382. Just cabbage? Not cucumber?

***That was an oversight on our part. We have revised that sentence to include both cabbage and cucumber. (line 406)***

Line 393. No discussion about Negativibacillus massiliensis and Mediterraneibacter glycyrrhizinilyticus

***Indeed, we were not able to discuss the results related to these two bacteria because there are no published data, to our knowledge, of their health implications. We added a paragraph to the discussion to describe this. The paragraph reads: “Findings from the LEfSe analysis indicated that Negativibacillus massiliensis was significantly enriched in group B at the end of the study, while Mediterraneibacter glycyrrhizinilyticus was significantly enriched in group C, at the end of the study. Unfortunately, there are no published data to date, describing the role of these bacterial species in human health; therefore, it is not known whether the changes observed in these bacteria in the present study have any implications to human health.” (line 422)***

Line 410-411. if it can affect individuals’ results even in the same group, why was it allowed for participants to choose one of those?

***The answer to this question is related to the justification for using two types of vegetables explained above.***
Line 416. What about analyzing with incremental data? Then there might be a change?

***Given that the primary aim of this study was to determine the feasibility of consumption of fermented vegetables, we are not sure that conducting additional analysis of this nature will provide any information that cannot be provided by an adequately powered trial. In fact, we just finished another trial where participants consumed only fermented cabbage versus no fermented cabbage for 8 weeks and we collected a total of 515 stool samples. We are currently analyzing the data for this trial and hope that we will be able to adequately address any changes in the gut microflora.***

* Editorial errors

Reviewer #2: A randomized pilot clinical trial was conducted which aimed to investigate the feasibility of regular consumption of fermented vegetables on inflammation and the composition of gut microflora. No significant changes in the levels of inflammatory markers were observed.

Minor revision:
Indicate the date range subjects were enrolled in the study.

***We modified the second sentence of the results to indicate dates of recruitment as well as dates of enrollment. (Lines 262 and 263)***

6. PLOS authors have the option to publish the peer review history of their article ([what does this mean?](https://nam10.safelinks.protection.outlook.com/?url=https%3A%2F%2Fjournals.plos.org%2Fplosone%2Fs%2Feditorial-and-peer-review-process%23loc-peer-review-history&data=04%7C01%7Ca.arikawa%40unf.edu%7C6adfd102344648aa459108d9eb24ba0f%7Cdf29b2fa8929482f9dbb60ff4df224c4%7C1%7C0%7C637799366464700793%7CUnknown%7CTWFpbGZsb3d8eyJWIjoiMC4wLjAwMDAiLCJQIjoiV2luMzIiLCJBTiI6Ik1haWwiLCJXVCI6Mn0%3D%7C3000&sdata=ICdfiB1olMu%2B8MQ7BPuX7eQlRM2Wf%2B4iItaqcywUwzg%3D&reserved=0)). If published, this will include your full peer review and any attached files.


If you choose “no”, your identity will remain anonymous but your review may still be made public.


**Do you want your identity to be public for this peer review?** For information about this choice, including consent withdrawal, please see our [Privacy Policy](https://nam10.safelinks.protection.outlook.com/?url=https%3A%2F%2Fwww.plos.org%2Fprivacy-policy&data=04%7C01%7Ca.arikawa%40unf.edu%7C6adfd102344648aa459108d9eb24ba0f%7Cdf29b2fa8929482f9dbb60ff4df224c4%7C1%7C0%7C637799366464700793%7CUnknown%7CTWFpbGZsb3d8eyJWIjoiMC4wLjAwMDAiLCJQIjoiV2luMzIiLCJBTiI6Ik1haWwiLCJXVCI6Mn0%3D%7C3000&sdata=zCEqFJoDTEojRJ3p2Y6f5Zcyk194kt4vlxNktvQbpos%3D&reserved=0).

Reviewer #1: No

Reviewer #2: No

[NOTE: If reviewer comments were submitted as an attachment file, they will be attached to this email and accessible via the submission site. Please log into your account, locate the manuscript record, and check for the action link "View Attachments". If this link does not appear, there are no attachment files.]

While revising your submission, please upload your figure files to the Preflight Analysis and Conversion Engine (PACE) digital diagnostic tool, [https://pacev2.apexcovantage.com/](https://nam10.safelinks.protection.outlook.com/?url=https%3A%2F%2Fpacev2.apexcovantage.com%2F&data=04%7C01%7Ca.arikawa%40unf.edu%7C6adfd102344648aa459108d9eb24ba0f%7Cdf29b2fa8929482f9dbb60ff4df224c4%7C1%7C0%7C637799366464700793%7CUnknown%7CTWFpbGZsb3d8eyJWIjoiMC4wLjAwMDAiLCJQIjoiV2luMzIiLCJBTiI6Ik1haWwiLCJXVCI6Mn0%3D%7C3000&sdata=A7DJ8WWaLjwqYreuGmCCFc2o3Q66gmy0unyBPlQTULM%3D&reserved=0). PACE helps ensure that figures meet PLOS requirements. To use PACE, you must first register as a user. Registration is free. Then, login and navigate to the UPLOAD tab, where you will find detailed instructions on how to use the tool. If you encounter any issues or have any questions when using PACE, please email PLOS at [figures@plos.org](mailto:figures@plos.org). Please note that Supporting Information files do not need this step.
