# Supplementary material for: The effects of fermented vegetable consumption on the composition of the intestinal microbiota and levels of inflammatory markers in women: A pilot and feasibility study
Source: PLoS One. 2022 Oct 6;17(10):e0275275. doi: 10.1371/journal.pone.0275275 (PMC9536613; doi:10.1371/journal.pone.0275275)
Supplement: Attachment — Submitted filename: Rebuttal letter.docx [file pone.0275275.s008.docx]

Rebuttal letter

Dear editor, please find below the responses to the comments by Reviewer #3.

Reviewer #3: Overall, there are some necessary data that are missing. These data must be included and discussed in the paper or the paper should not be accepted. The data that this reviewer suggest are:

1. Characteristics fermented (pickle) cabbage and cucumber should be presented and discussed. For example, microbiota of fermented (pickle) cabbage and cucumber, besides their lactic acid bacteria, pH, SCFA, and others. These data are very important to support the change of gut microbiota composition.

***We thank the reviewer for the important suggestion, and we believe that inclusion of these data greatly enhances our discussion. We included information about the composition of the vegetables used in the study in the results (lines 258-263)* and we also included another supplemental figure (S Figure 2) to illustrate the bacterial composition of the vegetables and expanded the discussion (lines 405-410).***

****Please note that all line numbers correspond to the manuscript with track changes version.***

2. Data on faecal quality (pH and consistency) are very important to be presented and discussed as it shows the direct impact of the intervention. This can also be done by adding the presence of viable microorganisms in products of each intervention.

***Unfortunately, we did not collect data on stool pH in our study. However, we added information about stool consistency to Table 2 and we also added a discussion about these findings (lines 376-386).***

3. Bloating (presented the intestinal disorder caused by diet intervention) must be recognized in the conclusion.

***We included a sentence in the conclusion recognizing that bloating is a possible side effect of regular consumption of fermented vegetables (lines 443-444).***

4. Line 326-327 - it was found that OTU3 (Faecalibacterium prausnitzii) was significantly enriched at week 6 in group A (P=0.022) – in this Figure 3, OTU3 does not clearly shows this bacterium has indeed significantly enriched in week 6. Since Faecalibacterium prausnitzii was mentioned in the conclusion as increased significantly, the data should be clear presented, as well as data on Ruminococcus torques.

***The findings regarding enrichment of F. prausnitzii were described in the manuscript but not accompanied by any figures. We agree with the reviewer that including a visual representation of this finding is important. Therefore, we decided to add a figure with the top 20 OTU which better represents the data described for changes in F. prausnitzii. We also performed additional analysis to compare the first 5 OTUs in Figure 3 within each treatment group. We have expanded the results to include these data (lines 313-317) and added a more robust discussion about these data (lines 422-436).***
