# Supplementary material for: The effects of fermented vegetable consumption on the composition of the intestinal microbiota and levels of inflammatory markers in women: A pilot and feasibility study
Source: PLoS One. 2022 Oct 6;17(10):e0275275. doi: 10.1371/journal.pone.0275275 (PMC9536613; doi:10.1371/journal.pone.0275275)
Supplement: Attachment — Submitted filename: Rebuttal letter.docx [file pone.0275275.s009.docx]

Rebuttal letter

Dear editor, please find below the responses to the minor revisions pointed out by Reviewer #2.

Reviewer #2: Minor revision:
1- Line 233: The Mann-Whitney test is generally referred to as the Mann-Whitney U test. **There is no mention of the Mann-Whitney test in our manuscript as this test is primarily used for comparisons between two independent groups and we wanted to compare three independent groups.**

2- Line 280: In addition to the means, include the corresponding standard deviations. **Standard deviations were included**

3- Line 304: Typographical error: Wilcoxon signed-rank test: **this error was corrected**

4- Table 3: Label the last column, p-value instead of p-trend. Technically the Kruskal-Wallis tests for differences among groups rather than testing for a trend. **Label was corrected**
